# Stimulus Uncertainty Affects Perception in Human Echolocation: Timing, Level, and Spectrum

**DOI:** 10.1037/xge0000775

**Published:** 2020-04-23

**Authors:** Liam J. Norman, Lore Thaler

**Affiliations:** 1 Department of Psychology, Durham University

**Keywords:** echolocation, blindness, sensory template, expectation

## Abstract

The human brain may use recent sensory experience to create sensory templates that are then compared to incoming sensory input, that is, “knowing what to listen for.” This can lead to greater perceptual sensitivity, as long as the relevant properties of the target stimulus can be reliably estimated from past sensory experiences. Echolocation is an auditory skill probably best understood in bats, but humans can also echolocate. Here we investigated for the first time whether echolocation in humans involves the use of sensory templates derived from recent sensory experiences. Our results showed that when there was certainty in the acoustic properties of the echo relative to the emission, either in temporal onset, spectral content or level, people detected the echo more accurately than when there was uncertainty. In addition, we found that people were more accurate when the emission’s spectral content was certain but, surprisingly, not when either its level or temporal onset was certain. Importantly, the lack of an effect of temporal onset of the emission is counter to that found previously for tasks using nonecholocation sounds, suggesting that the underlying mechanisms might be different for echolocation and nonecholocation sounds. Importantly, the effects of stimulus certainty were no different for people with and without experience in echolocation, suggesting that stimulus-specific sensory templates can be used in a skill that people have never used before. From an applied perspective our results suggest that echolocation instruction should encourage users to make clicks that are similar to one another in their spectral content.

The perceptual response to a sensory stimulus is not simply the result of a passive process. The brain in fact uses information from past sensory experiences to predict future stimuli and to modify its processing of those stimuli (Friston, 2005; Knill & Pouget, 2004). One way in which the brain does this is to use stimulus-specific sensory templates derived from past sensory experiences. At the neural level, these templates modulate the baseline activity of sensory neurons so that a stimulus can be processed with less neural activation if its evoked activity matches that of the template than if it does not (e.g., Alink, Schwiedrzik, Kohler, Singer, & Muckli, 2010; Kok, Mostert, & de Lange, 2017; Schröger, Marzecová, & SanMiguel, 2015). In human hearing, this means that an auditory stimulus can be detected with greater accuracy if the person knowns what to listen for, as well as when and where to direct their attention (Dau, Kollmeier, & Kohlrausch, 1997; Dau, Püschel, & Kohlrausch, 1996; Jones, Moynihan, MacKenzie, & Puente, 2002; Lawrance, Harper, Cooke, & Schnupp, 2014; Spence & Driver, 1994). The degree to which these templates can facilitate perception, however, is limited by how accurately and reliably they match the relevant target stimulus.

Echolocation is an acoustic method of sensing through sound reflections. Echolocation involves an initial emission that ensonifies the environment, with sound echoes being reflected back to the echolocator. Echolocation is mostly associated with bats and marine animals (Griffin & Galambos, 1941; Jones, 2005), but it is by now well-documented that people are capable of using echolocation, too (for reviews see Kolarik, Cirstea, Pardhan, & Moore, 2014; Norman & Thaler, 2017; Stoffregen & Pittenger, 1995; Thaler & Goodale, 2016). In echolocating bats it has been proposed that the neural pattern of responses to echoes is consistent with an auditory system that actively predicts echo responses (Simmons, 2012; Suga & Shimozawa, 1974). Also, on the behavioral level it has been shown that bats actively “steer” their emissions (e.g., modify beam direction and beam width; Kounitsky et al., 2015) and that they modify the spectral content of their emissions based on context (e.g., presence of absence of conspecifics or background noise; Amichai, Blumrosen, & Yovel, 2015) and behavioral goal (e.g., prey capture vs. landing; Boonman & Schnitzler, 2005; Schnitzler, Moss, & Denzinger, 2003), making them prime examples of active sensing systems.

In comparison, less is known about these effects in human echolocation. While humans show some evidence that they anticipate the sensory consequence of an echolocation emission (similar to bats; Suga & Shimozawa, 1974)—for example, by adjusting the intensity and number of clicks for weaker sound reflectors so as to boost signal-to-noise ratio (SNR; Thaler et al., 2018, 2019)—it is an open question whether their echolocation ability is actually improved when aspects of the echolocation sounds are predictable. Studying these effects in human echolocation offers a unique opportunity. Specifically, it will not only inform us of the perceptual mechanics of echolocation, but it also enables us to investigate the way in which stimulus-specific sensory templates can be used under novel conditions, that is, in a skill that people who are new to echolocation have never used before. More specifically, we can compare performance in people who are new to echolocation to performance in people who have extensive experience with this skill, that is, echolocation experts.

We gave participants an echo detection task and measured the effects of manipulating the certainty of an acoustic aspect of either the emission or the echo. This allowed us to directly compare listeners’ ability to detect the same echo under different conditions of stimulus predictability. We reason that an object will be detected with greater accuracy when a task-relevant property of either the emission or echo is certain compared to when it is uncertain. Furthermore, to address the potential role played by experience in echolocation, in all experiments reported here we tested blind expert echolocators (EEs) as well as blind and sighted controls (blind controls [BCs], and sighted controls [SCs], respectively). This allowed us to test whether any effects are dependent upon expertise in echolocation and/or blindness.

## Overview of Experiments

Binaural recordings of echolocation sounds were made, and participants then listened to these recordings through headphones and judged whether or not they heard an echo. In Experiments 1 and 2 we manipulated the certainty of the temporal onset of the emission (relative to when the listener pressed a button to initiate the emission) and/or the echo (relative to the emission; i.e., the emission-echo delay), respectively. In Experiments 3 and 4 we manipulated the certainty of the spectral content of the emission and/or the echo, respectively. In Experiments 5 and 6 we manipulated the certainty of the level of the emission and/or the relative level of the echo, respectively.

## General Methods

All experiments reported in this study shared some common elements, which are described below.

### Ethics

All procedures followed the British Psychological Society code of practice and the World Medical Association’s Declaration of Helsinki. The experiments received ethical approval from the Ethics Advisory Sub-Committee in the Department of Psychology at Durham University (approval number: Ref 14/13). All participants gave written informed consent to take part in this study. For blind participants, all materials were provided in accessible format and locations to sign were indicated through tactile markers.

### Participants

Three groups were tested. These were blind expert echolocators (EEs), blind controls (BCs), and sighted controls (SCs). EEs reported using click-based echolocation on a daily basis for more than 10 years. BCs and SCs reported having no prior experience with click-based echolocation, except for two of the BCs, who had taken part in a previous study in our lab requiring them to listen to echolocation sounds and to make clicks, but who did not meet our criteria for EEs in terms of regularity and duration of use of echolocation. All participants had normal hearing, as defined by pure tone audiometry thresholds <25 dB HL (measured with an Interacoustics AD629 audiometer using an automated Hughson-Westlake pure tone test), with the exceptions of BC8, BC10, BC11, and BC20 who had some hearing loss for frequencies above 4 kHz consistent with their age. Table 1 shows relevant details of the EEs and BCs who took part. Some participants took part in more than one of the experiments, as shown in Table 1. Participants were paid either at a rate of £10/hr or with the equivalent participant pool credit.^1^

### Apparatus and Recording Process

All sound recordings were made in a sound-insulated and sound-acoustic dampened room (approx. 2.9 m × 4.2 m × 4.9 m; 24-dBA noise floor) lined with foam wedges (cut-off frequency 315 Hz). Binaural sound recordings were made at a sampling rate of 96 kHz and resolution of 24 bits using a portable digital recorder (Tascam DR-100 MK2, TEAC Corporation, Japan) and in-ear microphones (Bruel & Kjaer Model 4101, Denmark). The microphones were placed either in the ears of a human subject (Experiment 1) or in the ears of a manikin^2^ (Experiments 2–6). In both cases, artificially generated emissions were played by a loudspeaker (Fostex FE103En) mounted on a metal pole (1-cm diameter) placed either at chest height of a human subject 33 cm from each ear (Experiment 1) or at the mouth of a manikin (Experiments 2–6). The loudspeaker was driven by a Dell Latitude E7470 laptop (Intel Core i56300U CPU 2.40 GHz, 8 GB RAM, 64-bit Windows 7 Enterprise) through a USB Soundcard (Creative Sound Blaster X-Fi HD Sound Card; Creative Technology Ltd., Creative Labs Ireland, Dublin, Ireland) and amplified by a Kramer 900N Stereo Power Amplifier (Kramer Electronics Ltd., Jerusalem, Israel). Sounds were played using Matlab R2015b (The Mathworks, Natick, MA). The level of amplification in all electronic equipment was held constant for the recording of all sounds across all conditions.[Table-anchor tbl1]

**Figure 1 fig1:**
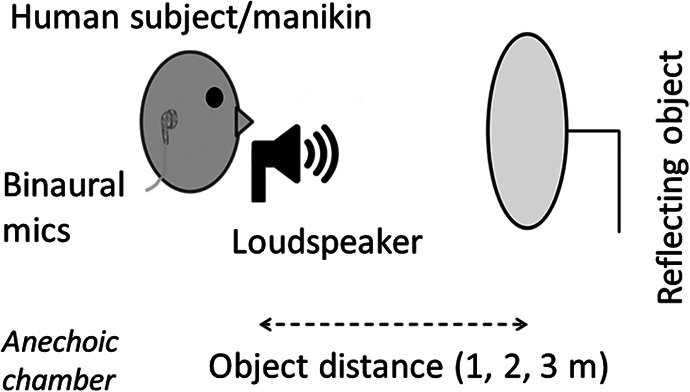
Sketch of the apparatus used for making the sound recordings. A human subject (Experiment 1) or a manikin (Experiments 2–6) was positioned behind a loudspeaker. The loudspeaker emitted either a click or noise burst. The loudspeaker was placed in front of the mouth (as shown here; Experiments 2–6) or in front of the chest (Experiment 1). A wooden disk (shown here; Experiments 2–6) or a plastic bowl (Experiment 1) was used as a reflecting object and positioned at a distance of 1, 2, or 3 m from the loudspeaker, or was not present at all. Recordings were made using binaural microphones.

In Experiment 1 the reflecting object was a plastic bowl (diameter 28 cm; depth 11 cm); in Experiments 2 to 6 it was a 0.8-cm thick wooden disk (50-cm diameter, made from plywood, double coated with matt emulsion paint). In each case, the object was vertically upright and mounted on a metal pole (1-cm diameter). The center of the object directly faced the loudspeaker (in Experiment 1, the open side of the bowl faced the loudspeaker), with the height of the object’s center matching that of the loudspeaker. The object could be absent, or present at either 1 m, 2 m, or 3 m. A single recording was made for each combination of object condition and emission condition (see details below). The setup of the recording apparatus is shown in Figure 1.[Fig-anchor fig1]

### Sound Emissions

Both clicks and 500-ms white noise bursts were used as emissions. For the click emissions, each click was constructed by first creating a 10-ms sinusoid (starting phase = 0°) of a desired frequency (either 3.5, 4.0, or 4.5 kHz; see Methods of individual experiments) and then multiplying all values up until the first half period of the sinusoidal wave by 0.6. The effect of this is that the early part of the sound has a lower level than the rest, but that there is still a sharp rise in sound intensity. This simulates the rising intensity of a natural click. Then, all values after the first 1.5 periods were multiplied by a decaying exponential function y = e^−6x^, where *x* is a series of linear equally spaced values between 0 and 1 that is equal in length to the number of values in the sinusoid between the first 1.5 periods of the sinusoidal wave and its end (at a sampling rate of 96 kHz). This effectively simulates the falling level of a natural click. This type of sound has been suggested to be a good approximation of the waveform created by a human echolocator’s mouth click (Martinez-Rojas, Hermosilla, Montero, & Espí, 2009; Thaler et al., 2017), and can be used by expert echolocators to detect objects with the same accuracy as when they use natural mouth clicks (e.g., Thaler & Castillo-Serrano, 2016).

The noise emissions were 500-ms bursts of noise either with a constant spectrum level (white noise; over the range of 0.2–20 kHz) or with a spectral variation (see Methods of individual experiments), created using Matlab R2015b (The Mathworks, Natick, MA). For noise with spectral variation, a 9-dB boost centered at a desired frequency (either 3.5, 4.0, or 4.5-kHz) was applied. The 9-dB boost was created by first filtering white noise with a Kaiser window bandpass filter (1-kHz wide passband centered at the desired frequency, with 0.1-kHz transition bands either side). This filtered noise was then added to unfiltered white noise, at a relative amplitude ratio of 3:1.

For Experiment 5, in which variation in the level of the emission was required, these variations were applied by digitally amplifying the emission sound by factors of 0 dB (i.e., baseline), −6 dB, and −12 dB (using the “Amplify” function in Audacity 2.1.2, 2016). Separate recordings of each of these emission sounds were then made.

### Sound Editing

Sound recordings were edited in Audacity (2.1.2, 2016), with specific editing steps described separately for each experiment where relevant, but some general steps are described here. Digital editing was used to equate the recorded sound level of the emissions (both clicks and noise) across variations in spectrum. Discrepancies in the recorded emissions arose because the beam pattern of the loudspeaker generating the emissions differed across frequency, in that more low-frequency than high-frequency sound energy traveled behind the loudspeaker. As we made our recordings using in-ear microphones placed behind the loudspeaker, this led to a lower intensity of higher-frequency emissions measured at the ear. To reduce the possibility that differences in the intensity of the emission would affect processing of the subsequent echo via forward masking/echo suppression (Litovsky, Colburn, Yost, & Guzman, 1999; Wallach, Newman, & Rosenzweig, 1949), we matched the intensity of the emissions across conditions. This was done by multiplying the 3.5- and 4.5-kHz emission recordings with scaling factors (i.e., single numerical values) in order to equate the peak intensity of the emission to that in the 4-kHz conditions (in the object-absent recordings). The “peak intensity” was defined as the single maximum absolute numerical value in the digital recording of the emission with no object present. This level correction does not guarantee an equal degree of forward masking across emission frequencies (e.g., Gockel, Moore, Patterson, & Meddis, 2003). Importantly, this has no bearing on our results because our measure of the effect of stimulus certainty always compared echo detection performance for the same sounds but under certain or uncertain conditions. Also, this correction did not necessarily equalize the peak intensity of the entire recording (emission + echo) because in some recordings the peak intensity of the echo was greater than that of the emission. With a target object of the size that we used, the peak intensity of the echo was greater than that of the emission at certain object distances. This is not unrealistic, as the emission and echo were recorded at the ear, with the loudspeaker positioned at the mouth facing away from the ear. The target object, on the other hand, directly faced the manikin. A final digital level correction was applied to all noise emission recordings to equate their peak recorded intensity to that of the click (both when no echo was present).

### Behavioral Paradigm

Participants were tested in the same sound-insulated and sound-acoustic dampened room in which the sound recordings had been made. Sounds were played through binaural in-ear headphones (Etymotic Research ER4B MicroPro) driven by a Dell Latitude E7470 laptop through a USB Soundcard (Creative Sound Blaster X-Fi HD Sound Card; Creative Technology Ltd., Dublin, Ireland). The sound file with the highest peak intensity was presented at 80 dB SPL (corresponding to the peak level of that sound). Participants sat upright and gave their response using a keyboard. Participants who were not fully blind wore a blindfold. All experiments were programmed in Matlab R2015b (The Mathworks, Natick, MA) and Psychtoolbox (v3.0.12; Brainard, 1997).

In each trial, participants were presented with a single sound that contained either an emission alone or an emission and an echo. After hearing the sound, they judged using a 6-point confidence rating scale (by pressing one of six keys on the keyboard) whether they heard the target object. Participants were not given detailed information about the target object, only that its presence could be detected by listening to the reflection of the click sound and that there were no other reflectors present. The scale ranged from 1 = *very confident object absent* to 6 = *very confident object present*. Participants received auditory feedback (50-ms tone) on each trial to indicate whether they were correct or not (1,500- or 900-Hz tone, respectively), with a rating of 1–3 being classed as a correct response for echo-absent trials and a rating of 4–6 being classed as a correct response for echo-present trials. Participants were instructed to prioritize accuracy over speed in their response.

In separate blocks, a property of the echolocation sounds was either certain or uncertain. In certain blocks, the property was constant from trial to trial. In uncertain blocks, the property was randomly determined from trial to trial (from a set of predefined values). In all experiments the order of these blocks was counterbalanced across participants.

### Calculating Sensitivity to the Echo

Sensitivity was calculated by tabulating the number of responses for each of the six confidence levels for object-absent trials and object-present trials, and using the software RScorePlus (Harvey, 2002) to fit a Gaussian unequal-variance signal detection model and derive a discriminability index (d_a_). d_a_ does not assume equal variance between the underlying *noise* and *signal + noise* Gaussian probability distributions, but is equivalent to *d*′ (d-prime) in the case of equal variance. A higher d_a_ indicates a greater sensitivity to the echo, and a d_a_ of zero indicates no sensitivity. Importantly, in all cases sensitivity was calculated for the same acoustic stimulus, but embedded either within certain or uncertain blocks of trials (for details see each experiment). In each experiment (unless stated otherwise), d_a_ values were first calculated separately for each permutation of factors for that experiment and then averaged across levels of object distance and levels of the relevant stimulus property (e.g., emission spectrum) to give a single measure of performance for each emission type under certain and uncertain stimulus conditions.

## Experiment 1: Temporal Onset of the Emission

In Experiment 1 we varied the certainty in the temporal onset of the emission, relative to a preceding button press. In source hearing, it has been shown that an auditory target is detected with greater accuracy when its temporal onset is known in advance, either because it is part of a periodic temporal sequence (Lawrance et al., 2014) or because its temporal onset can be predicted by a single preceding stimulus (Wright & Fitzgerald, 2004). If the same applies for echolocation, the detection of echoes should be more accurate when the onset of the emission is certain than when it is uncertain. We used both mouth clicks and longer noise bursts as emissions. In addition to single clicks, we used a train of four identical clicks to test whether any effects of certainty in temporal onset are dependent upon the sound being part of a predictable periodic sequence (as in Lawrance et al., 2014).

### Method

#### Participants

Three EEs (mean age = 42 years, *SD* = 8 years, one female), 10 BCs (mean age = 49 years, *SD* = 13 years, five females), and 24 SCs (mean age = 24 years, *SD* = 5 years, 19 females) took part. Table 1 shows details of individual participants.

#### Sound stimuli

**Figure 2 fig2:**
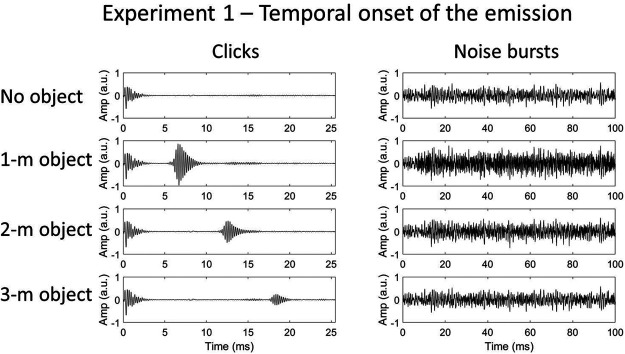
Waveforms of echolocation sounds used in Experiment 1. The clicks are shown in the left set of images, and the noise in the right set. From top to bottom: no object, 1-m object, 2-m object, and 3-m object. In the click recordings, the emission and echo are temporally separated, while for the noise recordings they temporally overlap due to the longer emission duration. Note that only a 100-ms sample of the noise emission recordings is shown here—the emission actually lasted 500 ms. The click-train stimuli (consisting of four consecutive clicks) are not shown in this figure. Note that the echo has a different envelope to the emission because the reflecting object in Experiment 1 was concave—a bowl. Here, and in later figures, the abbreviation a.u. refers to “arbitrary units.”

Recordings of clicks (4.0-kHz peak frequency) and white noise bursts were used as stimuli, as well as click trains consisting of four successive clicks (for more details see Task and Procedure section). The waveforms of the single click and noise burst recordings are shown in Figure 2 (click trains were simply multiple copies of the same single click recording).[Fig-anchor fig2]

#### Task and procedure

Blocks were defined by two factors: emission type (single click, click train, noise) and onset certainty (certainty, uncertainty). Each block contained 72 trials with one variable: object condition (absent, 1, 2, 3 m; in the ratio 3:1:1:1), presented in a random order. Participants pressed a key to begin each trial. In the certain blocks the sound played immediately. For the certain click train blocks the sound was played four times with a fixed interval between each sound (500 ms). In the uncertain blocks, a randomly selected delay of 0, 500, 1,000, 1,500, or 2,000 ms was introduced between the key press and the onset of the sound. For certain click train blocks, both the onset of the first click and the length of each interval between successive clicks were also randomly determined (equally likely to last 250, 375, 500, 675, or 750 ms). Two repetitions of each of these blocks were run across two separate testing sessions.

### Results and Discussion

**Figure 3 fig3:**
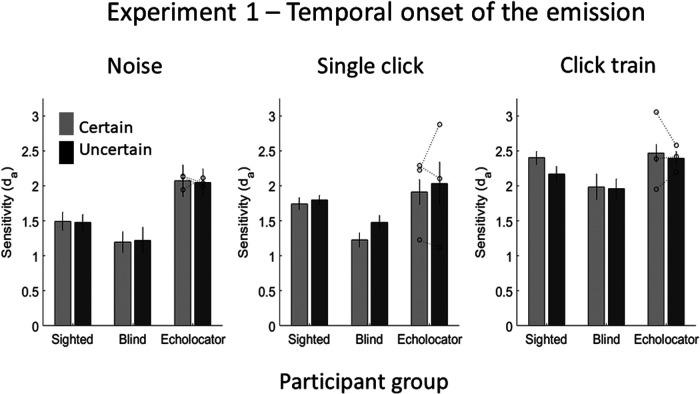
Results of Experiment 1, in which the certainty of the temporal onset of the emission was manipulated. Bars represent means and error bars represent the standard error of the mean (*SEM*) with between-subjects variance removed (Cousineau, 2005) in order to more accurately display the variance in the within-subject effect of stimulus certainty. Individual data points for each of the EEs (*n* = 3) are shown by the dotted lines overlaying the relevant bar graphs.

The d_a_ values (shown in Figure 3) were analyzed with a mixed-model analysis of variance (ANOVA), with the within-subject factors emission type and emission temporal onset certainty, and the between-subjects factor of participant group. Where sphericity could not be assumed, the Greenhouse-Geisser correction (*F*_GG_) was applied. There was a significant effect of emission type, *F*_GG_(1.517, 51.592) = 10.105, *p* = .001, with greater sensitivity for click train emissions (*M* = 2.2) than for single clicks (*M* = 1.7; *p* < .001) and noise (*M* = 1.6; *p* = .004).^3^ There was no effect of emission certainty *F*(1,34) = 0.014, *p* = .907, and no effect of participant group, *F*(2, 34) = 0.892, *p* = .419. There was no interaction between emission type and emission certainty, *F*(2, 68) = 1.079, *p* = .346. There was no interaction between emission type and participant group, *F*_GG_(3.035, 51.592) = 0.379, *p* = .771, between emission certainty and participant group, *F*(2, 68) = 0.523, *p* = .598, or between all three factors, *F*(4, 68) = 1.079, *p* = .346.[Fig-anchor fig3]

In addition to reporting group-level statistics, we performed statistical analyses to determine whether any of the EEs showed a significantly higher effect of stimulus certainty than the control participants. To do this, we first subtracted the mean d_a_ in the uncertain conditions from the mean d_a_ in the certain conditions. This gave a single measure for each participant, where a higher value represents a greater effect of stimulus certainty overall. The value calculated for each EE was then compared with the control group average (BCs and SCs collapsed into a single group) using a modified *t* test (Crawford & Garthwaite, 2002; Crawford & Howell, 1998), which tests whether a single case differs significantly from a control group. No individual EE was significantly higher on this measure compared with controls (EE1 = −0.0, *t*(33) = 0.067, *p* = .947; EE2 = −0.0, *t*(33) = 0.113, *p* = .911; EE3 = 0.0; *t*(33) = 0.039, *p* = .969; control group *M* = 0.0, *SD* = 0.4).

This experiment showed that participants—either EEs or controls—did not detect echoes more accurately when the temporal onset of the emission was certain than when it was uncertain. This was the case for all three emission types, which suggests that even when the stimulus is part of a predictable periodic sequence it is not detected with greater accuracy than when it is not. Due to the inclusion of three object distances in each block, the onset of the echo relative to the emission was certain. Importantly, however, this was the case in both the certain and uncertain blocks and the time window for those variations was much smaller than that used for the emissions. Based on these results, we conclude that humans do not improve their echo detection ability by forming sensory templates about when the emission is likely to occur.

Experiment 1 showed that participants detected the echo more accurately with the click train emission than with the other two. This can be explained by participants engaging in a process of averaging over multiple instances of a stimulus in order to maximize the signal-to-noise ratio, and thus improve target sensitivity—a strategy that humans use when actively echolocating (Thaler et al., 2018).

In natural active human echolocation, the echolocator always knows with certainty when the onset of the emission is to occur—that is one aspect of the emission that they are able to control. Nonetheless, our finding that performance was unaffected by the certainty of the emission appears contrary to results from perceptual judgments on nonecholocation sounds (e.g., Cravo, Rohenkohl, Wyart, & Nobre, 2013; Lawrance et al., 2014; Morillon, Schroeder, Wyart, & Arnal, 2016). The range of emission delays that we used in the uncertainty blocks (<2,000 ms) was equivalent to or even larger than those used in previous studies on source hearing (e.g., <350 ms, Lawrance et al., 2014; <770 ms, Morillon et al., 2016), so we are confident we used manipulations that have proven to be effective in the source-hearing context. One possible reason for this discrepancy is that in echolocation the relevant information is carried by the echo or the emission-echo relationship, and in Experiment 1 this information was uncertain under all conditions tested (because the echo was equally likely to come from an object at 1, 2, or 3 m on each trial, or to be absent altogether). It is possible that a more relevant temporal feature for echolocation is the emission-echo delay, and that humans would be affected by manipulations in the certainty in this property rather than simply in the emission onset. Experiment 2 tested this hypothesis.

## Experiment 2: Temporal Onset of the Echo

In Experiment 2, we varied the certainty in the temporal onset of the echo relative to the emission, but held constant the level of the emission and echo.

### Method

#### Participants

Three EEs (mean age = 36 years, *SD* = 13 years, all males), 10 BCs (mean age = 52 years, *SD* = 12 years, four females), and 24 SCs (mean age = 20 years, *SD* = 3 years) were tested. Table 1 gives details of individual participants.

#### Sound stimuli

**Figure 4 fig4:**
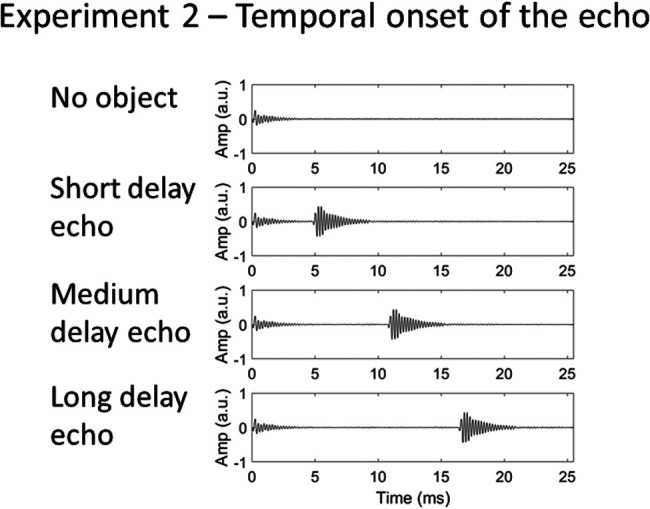
Waveforms of echolocation sounds used in Experiment 2, in which certainty in the echo’s temporal onset was varied. The emission was the same in all recordings.

Recordings of clicks (4.5-kHz peak frequency) were used as stimuli. We did not use the longer white noise burst emissions because for these sounds there was temporal overlap in recorded emission and echo. This would not allow us to manipulate echo delay without introducing secondary changes to the sounds. Sound recordings were digitally edited to produce three sound files in which the echoes differed only in temporal onset. To produce these, the echo produced by the object at a distance of 2 m was digitally cut and appended to the click emission at time points that correspond to the temporal onset of the echo from the object at 1 and 3 m, separately, thus leading to delays of ∼5, ∼11, and ∼17 ms, respectively. The point at which the echo waveform first rose above the noise floor was taken as the temporal onset of the echo. The waveforms of the three sounds are shown in Figure 4, along with that for the object absent.[Fig-anchor fig4]

#### Task and procedure

The certain blocks contained 20 trials with the echo absent or present with medium delay in the ratio 1:1. The uncertain blocks contained 60 trials with the echo absent or present with short, medium, or long delay in the ratio 3:1:1:1. Trials in each block were presented in a random order. Two repetitions of each block were run (giving a total of 20 trials for each certainty condition for the medium delay).

### Results and Discussion

**Figure 5 fig5:**
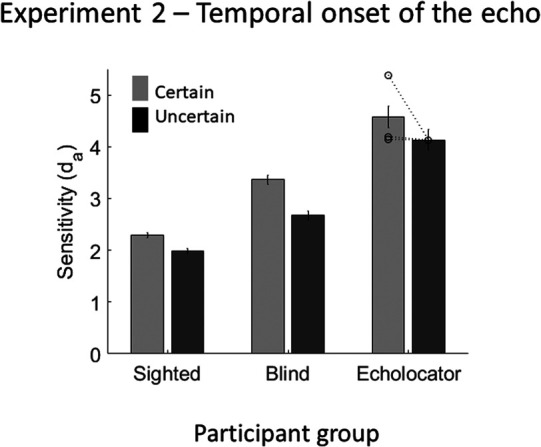
Results of Experiment 2, in which the certainty of the temporal onset of the echo was varied. Error bars represent (*SEM*) with between-subjects variance removed (Cousineau, 2005). Individual data points for each of the EEs (*n* = 3) are shown by the dotted lines overlaying the relevant bar graphs.

Sensitivity in the certain and uncertain conditions was calculated only for the medium-delay echo. The d_a_ values (shown in Figure 5) were analyzed with a mixed-model ANOVA, with the within-subject factor echo certainty, and the between-subjects factor participant group. There was a significant effect of echo certainty, *F*(1, 34) = 15.779, *p* < .001, with greater accuracy when the temporal onset of the echo was certain (*M* = 2.8) compared with uncertain (*M* = 2.4). There was a significant effect of participant group, *F*(2, 34) = 6.634, *p* = .004, EEs (*M* = 4.4) being more accurate than SCs (*M* = 2.2; *p* = .006) but not BCs (*M* = 3.0; *p* = .174). There was no significant interaction between echo certainty and participant group, *F*(2, 34) = 2.548, *p* = .093. Using a single measure of the effect of stimulus certainty on performance, where higher values indicate a greater overall effect of certainty, no individual EE measured significantly higher than controls (EE1 = 1.3, *t*(33) = 1.562, *p* = .128; EE2 = 0.1, *t*(33) = 0.664, *p* = .512, EE4 = 0.0; *t*(33) = 0.765, *p* = .450; control group *M* = 0.4, *SD* = 0.5).[Fig-anchor fig5]

At first glance, these results might suggest that people are sensitive to certainty in the temporal onset of an auditory stimulus on a much finer scale than previously found (e.g., approximately 76 ms in a task of comparative pitch judgment; Jones et al., 2002). Temporal coding for the onset of an echo in relation to an emission, however, is different from that for the onset of a single sound in isolation, due to the phenomenon of repetition pitch; two brief sounds separated by a short gap may be heard as a single sound with a pitch that is inversely related to the duration of the gap (Bilsen, 1966). The delay between two brief sounds might also determine the timbre of the overall sound—that is, the perceived property of a sound that is distinct from its level and pitch. People may, therefore, perform better when the echo’s onset is certain only because the perceived pitch or timbre of the overall sound is also certain.

In tasks of echo detection and discrimination, spectrum has been shown to be a useful cue (Schenkman & Nilsson, 2011), and in tasks of regular source hearing humans form sensory expectations for the spectrum of a sound (e.g., Greenberg & Larkin, 1968; Morillon et al., 2016). Experiment 3 therefore tested whether people are better at echo detection when the spectrum of the overall sound is certain than when it is uncertain.

## Experiment 3: Spectrum of the Emission

In Experiment 3, we varied the certainty in the peak frequency in the spectrum of the emission. The spectrum and level of the echo also varied with the spectrum of the emission, which is a natural effect of sound reflection with an object of the size we used.

### Method

#### Participants

Three EEs (mean age = 42 years, *SD* = 8 years, one female), 10 BCs (mean age = 50 years, *SD* = 12 years, six females), and 12 SCs (mean age = 24 years, *SD* = 5 years, nine females) took part. Table 1 gives details of individual participants.

#### Sound stimuli

**Figure 6 fig6:**
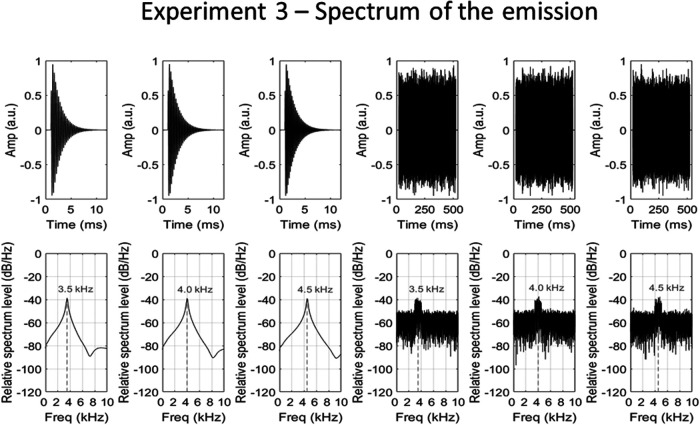
Waveforms and relative spectrum levels of the click emissions (left panels) and noise emissions (right panels) used in Experiment 3, in which the certainty of their spectrum was varied. The clicks were generated by modulating a sine wave of a particular frequency (3.5, 4.0, or 4.5 kHz) with a decaying exponential. The noise was generated by selectively adding a 9-dB boost (1-kHz bandwidth) to particular frequency components (3.5, 4.0, or 4.5 kHz) of “white” noise.

**Figure 7 fig7:**
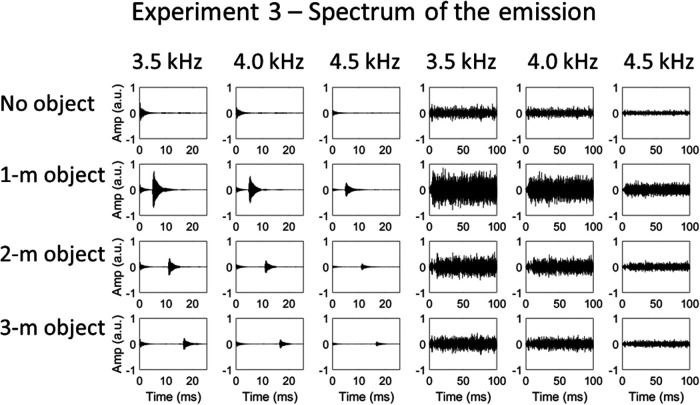
Waveforms of click (left panels) and noise (right panels) echolocation sounds used in Experiment 3, in which the certainty of the emission’s spectrum was varied. From top to bottom: no object, 1-m object, 2-m object, and 3-m object. From left to right (for each emission type): 3.5-, 4.0-, and 4.5-kHz emissions. Only a 100-ms sample of the noise emission recordings is shown here—the emission actually lasted 500 ms.

**Figure 8 fig8:**
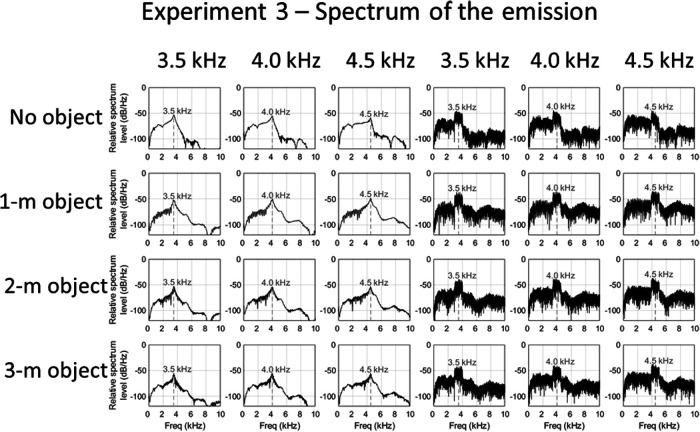
Relative spectrum levels of clicks (left panels) and noise (right panels) echolocation sounds used in Experiment 3, in which the certainty of the emission’s spectral content was varied. From top to bottom: no object, 1-m object, 2-m object, and 3-m object. From left to right (for each emission type): 3.5-, 4.0-, and 4.5-kHz emissions. Comb-filtering effects are visible in the panels when the object is present, which result from the interference caused by the temporal overlap of the emission and its echo.

Recordings of single clicks and noise bursts were used as stimuli. Three peak frequencies in the spectrum of the emissions were used: 3.5, 4.0, and 4.5 kHz. We chose these as they reflect a range that is found in natural human mouth clicks of EEs (Thaler et al., 2017; Zhang et al., 2017). The waveforms for all emissions, as well as their spectrum levels as a function of frequency, are shown in Figure 6. Waveforms of recorded emissions and echoes are shown in Figure 7. Relative spectrum levels of recorded emissions and echoes are shown in Figure 8.[Fig-anchor fig6][Fig-anchor fig7][Fig-anchor fig8]

#### Task and procedure

Blocks were defined by emission type (click, noise) and certainty. Each block contained 72 trials of two factors: peak frequency of the emission’s spectrum (3.5, 4.0, 4.5 kHz; in the ratio 1:1:1) and object condition (absent, 1, 2, 3 m; in the ratio 3:1:1:1). In the uncertain blocks, these 72 trials were presented in a random order in one run. In the certain blocks, they were presented in three miniblocks of 24, where each miniblock contained trials from only one of the three emission spectral frequencies. Thus, the same trials were used in the certain and uncertain blocks, and the blocks differed only in the order in which these trials were presented. Participants were informed in the short interval between miniblocks that the emission’s spectrum would be different in the upcoming miniblock. Six repetitions of each of these blocks were run across two separate testing sessions.

### Results and Discussion

**Figure 9 fig9:**
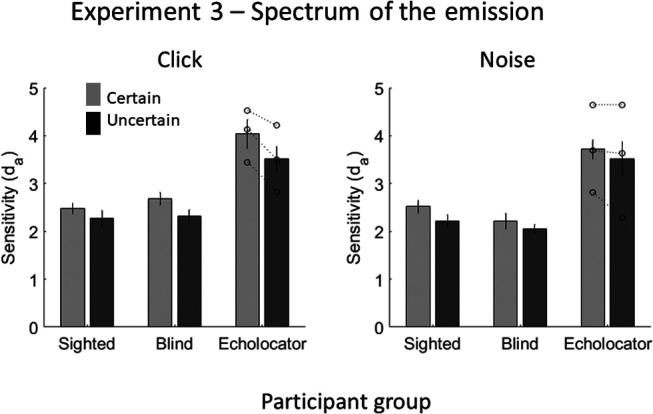
Results of Experiment 3, in which the certainty of the emission’s spectrum was manipulated. Error bars represent (*SEM*) with between-subjects variance removed (Cousineau, 2005). Individual data points for each of the EEs (*n* = 3) are shown by the dotted lines overlaying the relevant bar graphs.

d_a_ values were averaged across levels of emission spectrum as well as distance. The d_a_ values (shown in Figure 9) were analyzed with a mixed-model ANOVA, with the within-subject factors emission type and emission certainty and the between-subjects factor of participant group. There was a significant effect of emission certainty, *F*(1, 22) = 17.443, *p* < .001, accuracy being better when the spectrum of the emission was certain (*M* = 2.9) than when it was uncertain (*M* = 2.6). There was no significant effect of emission type, *F*(1, 22) = 0.788, *p* = .384, and no significant interaction between emission type and emission certainty, (*F*(1, 22) = 1.268, *p* = .272). There was a significant effect of participant group, *F*(2, 22) = 8.286, *p* = .002, EEs (*M* = 3.7) being more accurate than BCs (*M* = 2.3; *p* < .002) and SCs (*M* = 2.4; *p* = .003). Participant group did not significantly interact with emission certainty, *F*(2, 22) = 0.143, *p* = .868, or emission type, *F*(2, 22) = 0.499, *p* = .614. There was no significant interaction between emission certainty, emission type and participant group, *F*(2, 22) = 1.291, *p* = .295). Using a single measure of the effect of stimulus certainty on performance, where higher values indicate a greater overall effect, no individual EE measured significantly higher than controls (EE1 = 0.2, *t*(21) = 0.340, *p* = .737; EE2 = 0.6, *t*(21) = 1.046, *p* = .307, EE3 = 0.3; *t*(21) = 0.258, *p* = .799; control group *M* = 0.3, *SD* = 0.3).[Fig-anchor fig9]

These results suggest that humans improve their echo detection ability by forming sensory templates about the spectrum of the emission, and this is not dependent upon echolocation expertise or visual impairment. Similar effects have been observed for nonecholocation sounds, in that people make spectral predictions that lead to better performance (e.g., Morillon et al., 2016).

Emissions containing higher spectral frequencies lead to stronger echoes because, for an object of fixed proportions, sound composed of shorter wavelengths is more strongly reflected than sound with longer wavelengths.^4^ Thus, as emission spectral content varied in the previous experiment, so too did the level of the echo. Experiment 4 was designed to test whether people’s performance is affected specifically by certainty in the spectrum of the echo when the emission and echo levels remain constant.

## Experiment 4: Spectrum of the Echo

In Experiment 4, we varied the certainty in the peak frequency of the echo’s spectrum, but held constant the level of the echo and the spectrum and level of the emission.

### Method

#### Participants

Three EEs (mean age = 36 years, *SD* = 13 years, all males), 10 BCs (mean age = 56 years, *SD* = 11 years, five females), and 12 SCs (mean age = 24 years, *SD* = 6 years, nine females) took part. Table 1 gives details of individual participants.

#### Sound stimuli

**Figure 10 fig10:**
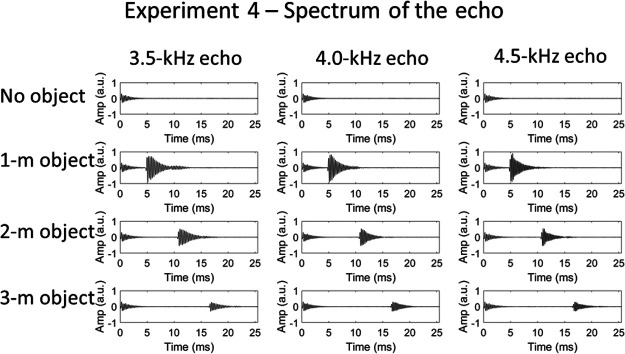
Waveforms of echolocation sounds used in Experiment 4, in which the certainty in the echo’s spectral content was varied. From top to bottom: no object, 1-m object, 2-m object, and 3-m object. From left to right: 3.5-, 4.0-, and 4.5-kHz echo. The emission was the same in all recordings (taken from the original 4.0-kHz emission recording).

**Figure 11 fig11:**
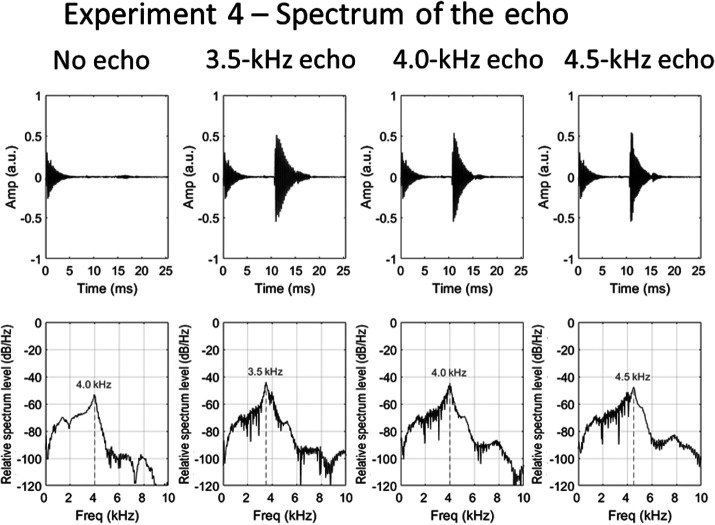
Waveforms (upper panels) and relative spectrum levels (lower panels of the echolocation sounds used in Experiment 4 (only object at 2 m is shown; objects at 1 and 3 m were also used)). From left to right: no echo, 3.5-kHz echo, 4.0-kHz echo, and 4.5-kHz echo. The emission was the same in all recordings (taken from the original 4.0-kHz emission recording). Comb-filtering effects are visible in the bottom panels when the object is present, which result from the interference caused by the temporal overlap of the emission and its echo.

Recordings of single clicks were used as stimuli. These were edited to generate three sound files for each object distance (1, 2, and 3 m), and one file for object absent. The three files for each object distance varied only in the frequency of the echo (the click emission was constant). In order to achieve this, the 4.0-kHz click emission was digitally cut from the object-absent recording and used as the emission in all sound files. Then, separately for each object distance, the echo from each of the three spectrum recordings (3.5, 4.0, and 4.5 kHz) was digitally cut and appended to the 4.0-kHz click emission at the appropriate time point for that object distance. The point at which the waveform first rose above the noise floor was taken as the temporal onset of the echo. This manipulation produced echolocation sounds in which the echo contained considerable energy at spectral frequencies for which there was much less energy in the emission. These sounds would be unlikely to arise in everyday situations, but they allowed us to test directly whether people are affected specifically by certainty in the peak frequency of the echo relative to that of the emission. To remove differences in the level of the echo that resulted from variations in the spectrum of the emission, the peak level of the 3.5- and 4.5-kHz echoes was equated to that of the 4.0-kHz echo for each object distance. Waveforms of the final recordings are shown in Figure 10. Figure 11 shows relative spectrum levels for the recordings for the object at 2 m.[Fig-anchor fig10][Fig-anchor fig11]

#### Task and procedure

Blocks were defined by object distance (1, 2, 3 m) and certainty. It was necessary to use only one object distance within a block to avoid variations in pitch that would arise from variations in the temporal delay between the click and echo, which might be misperceived as changes in the echo’s spectrum. Each block contained 60 trials of one variable: echo condition (absent, 3.5 kHz, 4.0 kHz, 4.5 kHz; in the ratio 3:1:1:1). In the uncertain blocks, these 60 trials were presented in a random order. In the certain blocks, they were presented in three miniblocks of 20, where each miniblock presented only echo-absent trials and echoes from only one of the three echo frequencies (in the ratio 1:1). Thus, the same trials were used in the certain and uncertain blocks, and the blocks differed only in the order in which these trials were presented. Participants were informed in the short interval between miniblocks that the echo’s frequency would be different in the upcoming miniblock of trials. Two repetitions of each of these blocks were run across two separate testing sessions.

### Results and Discussion

**Figure 12 fig12:**
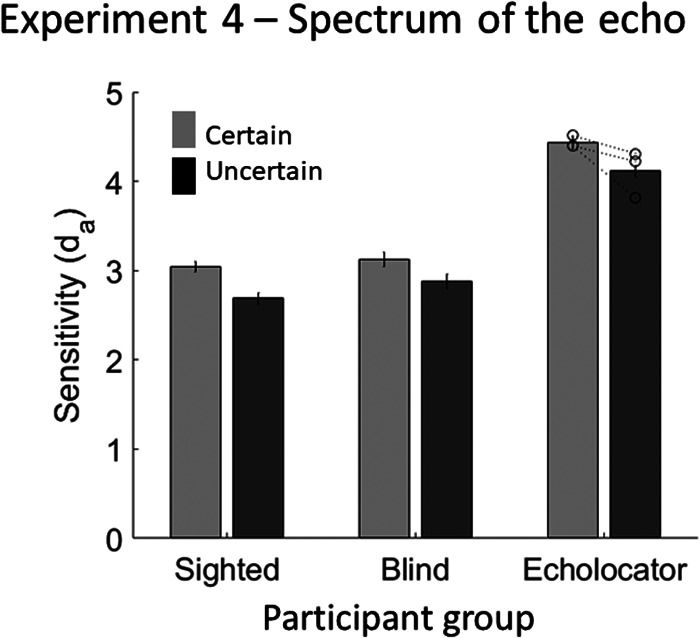
Results of Experiment 4, in which the certainty of the spectral content of the echo was varied. Error bars represent (*SEM*) with between-subjects variance removed (Cousineau, 2005). Individual data points for each of the EEs (*n* = 3) are shown by the dotted lines overlaying the relevant bar graphs.

d_a_ was not averaged across echo frequency—this factor was included in the analysis to test whether sensitivity depended on whether or not the emission and echo shared the same frequency. The d_a_ values (shown in Figure 12, but averaged across echo frequency) were analyzed with a mixed-model ANOVA, with the within-subject factors echo certainty and echo spectrum, and the between-subjects factor participant group. There was a significant effect of certainty, *F*(1, 22) = 8.187, *p* = .009, with greater accuracy when the spectrum of the echo was certain (*M* = 3.2) than when it was uncertain (*M* = 2.9). There was a significant effect of participant group, *F*(2, 22) = 4.633, *p* = .021, with EEs (*M* = 4.3) being more accurate than BCs (*M* = 3.0; *p* < .043) and SCs (*M* = 2.9; *p* = .019). There was no significant interaction between echo certainty and participant group, *F*(2, 22) = 0.140, *p* = .870. There was no significant effect of echo frequency, *F*(2, 44) = 1.263, *p* = .293, and no significant interaction between echo frequency and any of the other variables (Frequency × Certainty: *F*(2, 44) = 0.056, *p* = .945; Frequency × Participant Group: *F*(4, 44) = 0.291, *p* = .882; Frequency × Certainty × Participant Group: *F*(4, 44) = 0.734, *p* = .574). Using a single measure of the effect of stimulus certainty on performance, where higher values indicate a greater overall effect, no individual EE measured significantly higher than controls (EE1 = 0.6, *t*(21) = 0.594, *p* = .559; EE2 = 0.2, *t*(21) = 0.224, *p* = .825; EE4 = 0.2; *t*(21) = 0.286, *p* = .778; control group *M* = 0.3, *SD* = 0.5).[Fig-anchor fig12]

These results suggest that humans improve their echo detection ability by forming sensory templates specifically about the echo’s spectrum, and this is not dependent upon echolocation expertise and/or visual impairment. This is consistent with the hypothesis that spectrum is a task-relevant quality for detecting objects through sound echoes (Schenkman & Nilsson, 2011). One other factor that is relevant to echo detection is sound level. People are better at detecting echoes of higher sound level, regardless of the spectral content of the sound (Norman & Thaler, 2018), and people produce more intense emissions for weaker target reflectors, suggesting that increasing the level of the emission is part of an adaptive process to increase SNR (Thaler et al., 2019, 2018). Furthermore, Flanagin et al. (2017) have shown that the level of the emission is related to an echolocator’s ability to judge room size. Experiment 5 was designed to assess whether people’s echo detection ability is affected by the certainty of the level of echolocations sounds. The experiment was directly analogous to Experiment 3 in terms of its design, methods, and results analysis, but here variations in the emission’s level were used instead of spectral content.

## Experiment 5: Level of the Emission

In Experiment 5, we varied the certainty of the level of the emission. The level of the echo varied with the level of the emission, which is a natural effect of sound reflection.

### Method

#### Participants

Three EEs (mean age = 42 years, *SD* = 8 years, one female), 10 BCs (mean age = 49 years, *SD* = 12 years, five females), and 12 SCs (mean age = 24 years, *SD* = 5 years, 10 females) took part. Table 1 gives details for individual participants.

#### Sound stimuli

**Figure 13 fig13:**
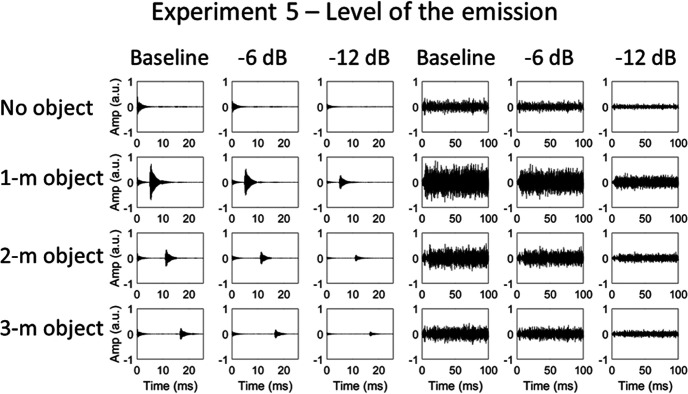
Waveforms of the click (left panels) and noise (right panels) echolocation sounds used in Experiment 5, in which the certainty of the emission level was manipulated. From left to right (for each emission type): baseline level, −6 dB level, and −12 dB level emissions. From top to bottom: object absent, object at 1, 2, and 3 m distance. Note that only a 100-ms sample of the noise emission recordings is shown here—the emission actually lasted 500 ms.

Clicks with a peak frequency of 4.5 kHz and white noise were used as the emissions. Three values of the emissions’ level were used: 0 dB (baseline), −6 Db, and −12 dB. These values refer to the level of digital amplification that was applied to the emission used in the recording (see Sound Emissions section in General Methods). These level differences are well above the normal just-noticeable differences (JNDs) for level discrimination in humans (1–2 dB at moderate levels under optimal conditions; Dykstra, Koh, Braida, & Tramo, 2012; Yost, 2006). Waveforms of emissions (and echoes) are shown in Figure 13.[Fig-anchor fig13]

#### Task and procedure

The task and procedure were the same as for Experiment 3, the only difference being that blocks of trials were defined based on the certainty of the emission’s level, rather than spectral content.

### Results and Discussion

**Figure 14 fig14:**
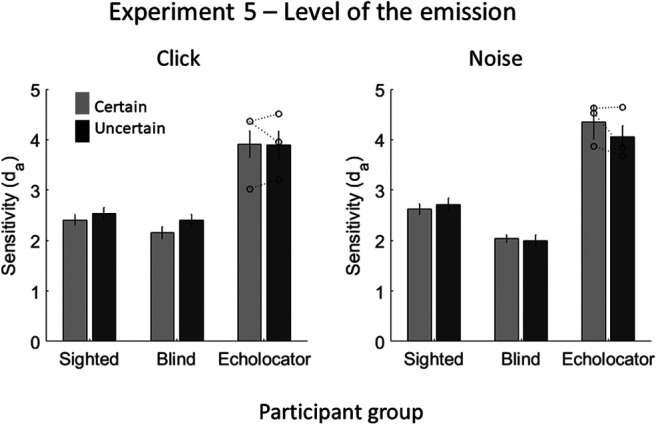
Results of Experiment 5, in which the certainty of the level of the emission was varied. Error bars represent (*SEM*) with between-subjects variance removed (Cousineau, 2005). Individual data points for each of the EEs (*n* = 3) are shown by the dotted lines overlaying the relevant bar graphs.

The d_a_ values (shown in Figure 14) were entered into a mixed-model ANOVA, with the within-subject factors emission type and emission certainty, and the between-subjects factor of participant group. There was no significant effect of emission certainty, *F*(1, 22) = 0.049, *p* = .827, or of emission type, *F*(1, 22) = 0.306, *p* = .586, and no significant interaction between the two, *F*(1, 22) = 1.751, *p* = .199. There was a significant effect of participant group, *F*(2, 22) = 13.605, *p* < .001, EEs (*M* = 4.1) being more accurate than BCs (*M* = 2.1; *p* < .001) and SCs (*M* = 2.6; *p* = .001). Participant group did not significantly interact with emission certainty, *F*(2, 22) = 0.651, *p* = .531, or emission type, *F*(2, 22) = 2.056, *p* = .152. There was no significant interaction between emission certainty, emission type and participant group, *F*(2, 22) = 0.475, *p* = .628. Using a single measure of the effect of stimulus certainty on performance, where higher values indicate a greater overall effect, no individual EE was significantly higher compared to controls (EE1 = 0.2, *t*(21) = 0.778, *p* = .446; EE2 = 0.0, *t*(21) = 0.323, *p* = .750; EE3 = 0.2; *t*(21) = 0.922, *p* = .367; control group *M* = −0.1, *SD* = 0.4).[Fig-anchor fig14]

These results suggest that humans do not improve their echo detection ability by forming sensory templates about the level of the emission. This was true for both clicks and noise bursts and, although EEs were better able to detect the echo than BCs and SCs, there was no effect of the certainty of the emission for any group. It is unlikely that this null finding arose because the levels used were not discriminable by participants—JNDs for level changes are much smaller than the stimulus difference used here. It should be noted, however, that for brief transient sounds, like clicks, level discrimination is poorer than for longer stimuli (Raab & Taub, 1969). Nonetheless, we found no effect of the certainty of the level of 500-ms noise bursts.

In echolocation, the relevant information is carried by the relationship between emission and echo. Experiment 6 was therefore designed to test this whether people’s ability to detect an echo is affected by the certainty of the level of the echo relative to that of the emission.

## Experiment 6: Relative Level of the Echo

In Experiment 6, we varied the certainty in the level of the echo, but held constant the level of the emission.

### Method

#### Participants

Experiment 6 was run in the same testing session as Experiment 2, and used the same participants.

#### Sound stimuli

**Figure 15 fig15:**
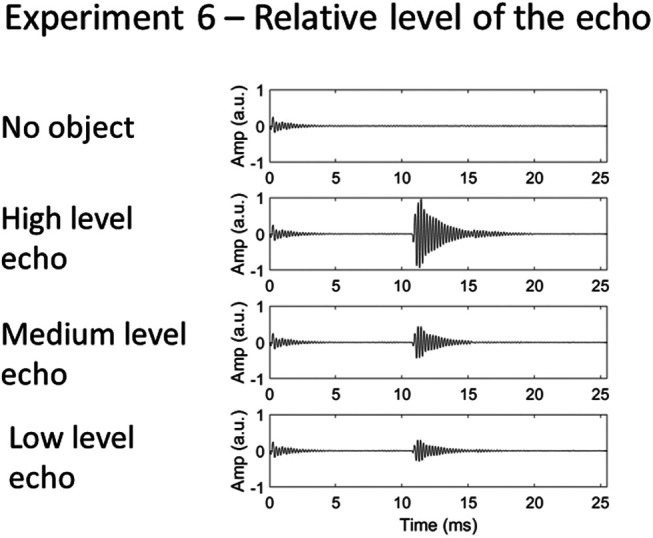
Waveforms of echolocation sounds used in Experiment 6, in which the certainty of the echo’s level was varied. The emission was the same in all recordings (taken from the original −6-dB level emission recording).

Recordings of clicks (4.5-kHz peak frequency) were used as stimuli. The recordings were digitally edited to produce three sound files in which the echoes differed only in their level. To produce a set of sounds that varied in the echo’s level, the echo from each of the three object distances from the −6-dB emission level recordings was digitally cut. These echoes were then each appended to the click emission from the −6-dB level recording at the time point corresponding to the temporal onset of the echo from the object present at 2 m. Thus, three sound files were created in which the level of the echo varied, but its temporal onset was constant. The waveforms of these sounds are shown in Figure 15. The difference in level between the high and medium level echoes was 6.8 dB, and that between the high and low level echoes 10.2 dB.[Fig-anchor fig15]

#### Task and procedure

Blocks were defined by one factor—echo level certainty. The certain blocks contained 20 trials of one variable: echo condition (absent, present; in the ratio 1:1). Only the medium level echo was used in the certain blocks. The uncertain blocks contained 60 trials split by the factor echo condition (absent, low level, medium level, high level in the ratio 3:1:1:1). Trials in each block were presented in a random order. Two repetitions of each block were run.

### Results and Discussion

**Figure 16 fig16:**
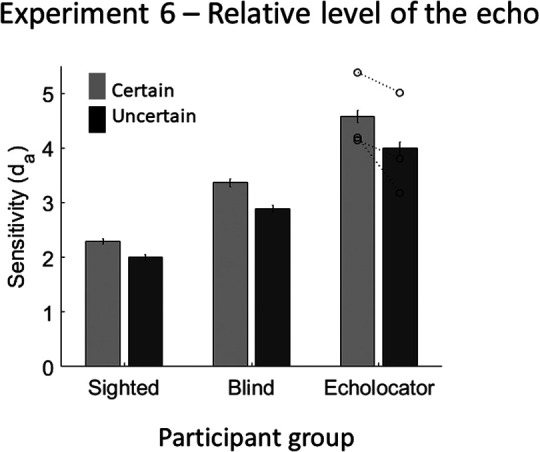
Results of Experiment 6, in which the certainty of the level of the echo was varied. Error bars represent (*SEM*) with between-subjects variance removed (Cousineau, 2005). Individual data points for each of the EEs (*n* = 3) are shown by the dotted lines overlaying the relevant bar graphs.

d_a_ was calculated only for the medium level echo in both the certain and uncertain blocks, because this was the only level that was included in the certain blocks. The d_a_ values (shown in Figure 16) were entered into a mixed-model ANOVA, with the within-subject factor echo certainty and the between-subjects factor participant group. There was a significant effect of certainty, *F*(1, 34) = 16.978, *p* < .001, accuracy being greater when the level of the echo was certain (*M* = 2.8) than when it was uncertain (*M* = 2.5). There was a significant effect of participant group, *F*(2, 34) = 6.189, *p* = .005), EEs (*M* = 4.3) being more accurate than SCs (*M* = 2.2; *p* = .006) but not BCs (*M* = 3.1; *p* = .334). There was no significant interaction between certainty and group, *F*(2,34) = 1.135, *p* = .333. Using a single measure of the effect of stimulus certainty on performance, where higher values indicate a greater overall effect, no individual EE was significantly higher compared with controls (EE1 = 0.4, *t*(33) = 0.062, *p* = .951; EE2 = 1.1, *t*(33) = 1.400, *p* = .172; EE4 = 0.3; *t*(33) = 0.015, *p* = .988; control group *M* = 0.3, *SD* = 0.5).[Fig-anchor fig16]

These results suggest that humans improve their echo detection ability by forming sensory templates about the level of the echo relative to that of the emission, and this is not dependent upon echolocation expertise or visual impairment.

## General Discussion

One way in which the brain may improve processing of sensory input is to form stimulus-specific sensory templates. The accuracy and reliability of these templates are partly determined by the certainty with which the properties of the target stimulus can be estimated from past sensory experiences (e.g., Alink et al., 2010; Dau et al., 1996, 1997; Jones et al., 2002; Kok et al., 2017; Lawrance et al., 2014; Schröger et al., 2015). In order to understand how this might apply in human echolocation, we manipulated the certainty of properties of either the emission or echo in an echo detection task. Previous work has shown that echolocation experts adjust the intensity or number of mouth clicks in order to suit different conditions (Thaler et al., 2019, 2018), but the present study is the first to show that echolocation ability is actually better when properties of the echolocation sounds are certain.

We found that people detected an echo with higher accuracy when the echo (relative to the emission) was certain either in its temporal onset, spectral content or level, when the emission was held constant. In contrast, when the certainty of the overall sound was manipulated (i.e., emission and echo together), accuracy was higher only when the spectrum was certain, but there was no effect of certainty for emission temporal onset or level. These results are consistent with the notion that the most important information for echolocation is carried by the acoustic relationship between the emission and echo, as this information indicates the reflective properties of the object, as opposed to the overall level of the sound. Under conditions of certainty, stimuli induce sensory templates that are measurable in neural activity, and the strength of this neural activity positively predicts performance on a behavioral task (e.g., Kok et al., 2017). Based on this previous work and others (e.g., Dau et al., 1996, 1997), we reason that participants formed internal sensory templates of the object-absent and object-present echolocation sounds that could be compared with the actual sound heard on each trial. This is a plausible mechanism by which participants’ sensitivity to the echo was affected by our manipulations of stimulus certainty. One additional explanation is that stimulus certainty allowed participants to *predictively code* the relevant stimulus features. Previous research has shown that auditory cortical activity is determined both by stimulus *predictions* as well as stimulus input (Heilbron & Chait, 2018; Rubin, Ulanovsky, Nelken, & Tishby, 2016), but the design of our experiments did not allow us to test whether our effects were the result of stimulus predictions. Therefore, the use of a sensory template that is, “knowing what to listen for,” remains the most parsimonious and sufficient framework by which to explain our results. Future research could address the specific role played by predictive mechanisms in human echolocation.

On a broad level, our results are in line with previous studies with nonecholocation sounds, which have shown that a person’s ability to detect or discriminate sounds is improved when certain aspects of those sounds are certain (e.g., Jones et al., 2002; Lawrance et al., 2014; Morillon et al., 2016; Rimmele, Jolsvai, & Sussman, 2011). There are, however, some important differences. Specifically, in Experiment 1 we found no evidence that the certainty of the temporal onset of the emission, and thus the overall sound, affected performance (even for click-train stimuli). This is contrary to findings for nonecholocation sound, where it has been consistently shown that a sound is processed more accurately when its onset is certain (e.g., Jones et al., 2002; Lawrance et al., 2014; Morillon et al., 2016; Ng, Schroeder, & Kayser, 2012; Rimmele et al., 2011; Schmidt-Kassow, Schubotz, & Kotz, 2009; although see Zoefel & Heil, 2013). Thus, our findings suggest that these effects might manifest differently for auditory tasks involving echolocation compared with those that do not, and this might be determined by the differential prioritization of acoustic cues based on their relevance to each task.

One acoustic cue that is relevant in tasks of echo detection is the emission-echo delay, and in Experiment 2 we observed an effect of stimulus certainty for a small range of emission-echo delays (approximately 12 ms). Previous studies have only shown behavioral effects with temporal windows of greater length. For example, participants were significantly less accurate in a pitch comparison task (Jones et al., 2002) when the onset of the comparison tone was earlier or later than predicted by 76 ms. Effects of the temporal certainty relating to the emission-echo delay, however, might be fundamentally different to those for nonecholocation sounds, especially when the delay is short. Instead of encoding the relative temporal onset of the echo, people might instead encode the perceived pitch or timbre that results when two brief sounds are separated by a short gap (repetition pitch; Bilsen, 1966). Although people are capable of detecting gaps as brief as 2 to 3 ms in sounds that are broadband and significantly above threshold (Plomp, 1964), there has been no evidence that they are affected by the temporal certainty of a stimulus over such a short scale. Further work is therefore needed to determine the mechanism behind the effect we observed. On a related note, previous work has shown that blind people are more accurate in an auditory gap detection task than sighted people (Muchnik, Efrati, Nemeth, Malin, & Hildesheimer, 1991). We found no difference between blind and sighted participants in the temporal certainty effects we observed, suggesting that any individual differences with respect to auditory temporal resolution do not explain the effect of stimulus certainty on performance.

The effect of certainty in the spectral content of the emission found in Experiment 3 may have arisen because emissions that vary in spectrum can produce complex secondary variations in the reflected sound (i.e., variations in echo level for an object of fixed proportions; Norman & Thaler, 2018), in comparison to variations in level or temporal onset. In order to minimize the effects of those secondary variations, it might be advantageous for the brain to take into account the spectrum of the emission. Yet, even when we held the spectral content of the emission constant, and varied only the spectral content of the echo (without any variation in level; Experiment 4), participants performed better when the spectral content of the echo was certain. This suggests that important information is carried by the spectrum of the echo and/or the spectral relationship between click and echo. Overall, the spectral effects we observed are in agreement with previous suggestions that spectral composition is an important perceptual feature for echolocation in humans (Schenkman & Nilsson, 2011).

With respect to the level of the sounds, we found that certainty of the level of the overall sound (emission + echo; Experiment 5) did not affect performance. This is comparable with the results of a previous study suggesting that sound level was not a relevant acoustic feature for echolocation (Schenkman & Nilsson, 2011). We did find, however, that certainty of the level of the echo *relative* to the emission did affect performance (Experiment 6)—a result that has not been shown previously—which suggests that this is a property that is relevant for echo detection and is incorporated into the formation of stimulus-specific sensory templates to improve sensitivity.

The effects of stimulus certainty that we found were not dependent upon echolocation expertise or visual impairment. Although echolocation experts did better overall than control participants, echolocation expertise did not interact with stimulus certainty. Remarkably, this implies that people form stimulus-specific sensory templates for echolocation even if this is a skill that they have never used before. Such a result lends support to the notion that the human brain is naturally able to support echo detection, regardless of whether the individual has developed it as an expert skill. A previous study, for example, showed that the same functional dissociation in the brain for the processing of motion conveyed through source sounds compared with echo sounds was present in both echolocation experts and sighted echo beginners (Thaler, Milne, Arnott, Kish, & Goodale, 2014). This might explain why people who are new to echolocation are able to pick up fundamental abilities such as echo detection very quickly.

The sizes of the effects that we observed were modest (approximately 13% increase in participant sensitivity for certain compared to uncertain conditions), compared with those found in some previous studies with near-threshold targets (e.g., 46%; Lawrance et al., 2014). In other studies, however, the effect has been a similar size to ours (e.g., approximately 10%; Jones et al., 2002). We expect that the effect size could be increased by greater task difficulty or by increasing the range of the relevant stimulus property in the uncertain conditions. These effects are likely to be task-specific, and may not necessarily apply in the same way to aspects of echolocation other than echo detection (e.g., ranging or localization). Some studies, for example, have identified separate templates that compete with one another based on task demands (e.g., Morillon et al., 2016).

To conclude, we have shown that a person’s ability to detect an echo is improved when certain properties of either the emission or echo are certain. We reason that this is because stimulus certainty allows the echolocator to form better-defined sensory templates against which the actual sensory input can be compared. Similar effects have been previously shown in bats (e.g., Suga & Shimozawa, 1974), and even though very different paradigms have been used, similar processing principles may apply across species. We suggest that future research in this area would benefit from using both human and nonhuman (e.g., bats) models.

Finally, echolocation can be a useful tool for people who are blind because it provides them with additional sensory information about their distal environment and it can have benefits for their mobility (Thaler, 2013). The current results with respect to the effects of emission spectral content suggest that echolocation instruction should encourage users to make clicks that are spectrally similar to one another.

## Figures and Tables

**Table 1 tbl1:** Details of 4 EEs and 20 BCs

Expert echolocators						Experiment number					
ID	Gender	Age	Degree of vision loss	Cause and onset of vision loss	Echolocation use	1	2	3	4	5	6
EE1	M	50	Total blindness.	Birth; retinoblastoma; enucleation at 13 months.	Daily; since early childhood/no exact age remembered	x	x	x	x	x	x
EE2	M	35	Total blindness.	Gradual sight loss since birth due to glaucoma.	Daily; since 12 years old	x	x	x	x	x	x
EE3	F	41	Total blindness.	Birth; retinoblastoma; enucleation at 22 months.	Daily; since 30 years old	x		x		x	
EE4	M	24	Total blindness.	Sudden loss of vision at age 12 (cause unknown); enucleation at age 19 (to alleviate ocular discomfort).	Daily; since 12 years old		x		x		x
Blind controls											
BC1	M	67	Residual bright light perception.	Leber’s Amaurosis; from birth.	None	x	x		x		x
BC2	F	27	Total blindness in right eye; approximately 1 degree of visual field in the left.	Leber’s Amaurosis; from birth. Cataracts (with increasing severity); from birth.	None			x		x	
BC3	M	32	Total blindness.	Retinopathy of prematurity. Some vision in right eye from birth; retinal detachment in right eye at age 12.	None	x	x	x		x	x
BC4	F	56	Tunnel vision in left eye.	Accident resulting in damage to optic nerve/optic chiasm, age 44.	None			x	x	x	
BC5	M	63	Central vision in right eye; residual bright light perception in both eyes.	Glaucoma. Poor vision since birth, with increasing severity; registered blind age 50.	None		x				x
BC6	F	59	Total blindness in left eye; peripheral vision in right eye.	Stichler’s syndrome; retinal sciasis; from birth with increasing severity.	None	x	x		x		x
BC7	M	53	Residual bright light perception.	Retinitis pigmentosa; official diagnosis age 10. Gradual sight loss from birth.	Some experience; very little regular use		x			x	x
BC8	M	69	Residual bright light perception; some shape perception	Retinal dystrophy (exact cause unknown); official diagnosis age 6–7.	None				x		
BC9	F	64	Total blindness.	Undeveloped iris; from birth.	None	x	x	x	x	x	x
BC10	F	56	Total blindness in left eye; residual bright light perception and some shape perception in right eye.	Coloboma; from birth.	Some experience; very little regular use	x			x		
BC11	F	52	Residual bright light perception.	Optic nerve atrophy; age 5.	None	x					
BC12	F	46	Total blindness.	Coloboma; from birth. Some sight in right eye until age 18.			x	x			x
BC13	F	51	Total blindness.	Cone-rod dystrophy; cataracts; retinal vessel problems; official diagnosis age 6. Total blindness at age 41.	None		x	x		x	x
BC14	F	62	Residual bright light perception.	Retinal development abnormality; from birth.	None			x		x	
BC15	M	70	Residual bright light perception.	Unknown cause; from birth.	None			x			
BC16	M	45	Total blindness.	Ocular albinism. Gradual sight loss from birth.	No regular use	x	x	x	x	x	x
BC17	M	45	Total blindness.	Blood clot damaging optic nerve; age 15.	No regular use	x		x	x	x	
BC18	F	36	Residual bright light perception.	Unknown cause; from birth.	None	x			x		
BC19	M	37	Tunnel vision in both eyes.	Retinitis pigmentosa; gradual from birth; official diagnosis age 13.	None	x	x				x
BC20	M	58	Total blindness.	Birth; retinoblastoma; enucleation at 2 years.	None				x	x	
*Note*. The experiments that each participant took part in are marked with “x” in the rightmost columns. EE = expert echolocators.											

## Footnotes

1 Participant pool credit is given to psychology undergraduate students as part of an internal departmental system for recruiting participants for experiments. One credit is given for 1 hr of participation. Students who collect enough credit can use the participant pool resource for their own recruitment.

2 The manikin was custom-made, consisting of a torso and head made of high-density foam covered with soft plastic having a skin-like texture. Anthropometric details of this manikin have been published elsewhere (Norman & Thaler, 2018).

3 All post-hoc tests reported are the result of Bonferroni-adjusted pairwise comparisons.

4 This depends on the surface properties of the reflecting object.
